# Sleep, chronotype, and sleep hygiene in children with attention-deficit/hyperactivity disorder, autism spectrum disorder, and controls

**DOI:** 10.1007/s00787-017-1025-8

**Published:** 2017-07-08

**Authors:** K. B. van der Heijden, R. J. Stoffelsen, A. Popma, H. Swaab

**Affiliations:** 1 Department of Clinical Child and Adolescent Studies, Leiden University, Wassenaarseweg 52, Box 9555, 2300 RB Leiden, The Netherlands; 2 Leiden Institute for Brain and Cognition, Leiden University, Leiden, The Netherlands; 3 Department of Child and Adolescent Psychiatry, VU University Medical Center/De Bascule, Amsterdam, The Netherlands; 4 Institute for Criminal Law and Criminology, Leiden University, Leiden, The Netherlands

**Keywords:** ADHD, Autism spectrum disorder, Sleep, Chronotype, Children, Sleep hygiene

## Abstract

Sleep problems are highly prevalent in ADHD and autism spectrum disorder (ASD). Better insight in the etiology is of clinical importance since intervention and prevention strategies of sleep problems are directed at underlying mechanisms. We evaluated the association of sleep problems and sleep patterns with sleep hygiene (behavioral/environmental practices that influence sleep quality, e.g. caffeine use), access to electronic media, chronotype, and anxiety/depression in children aged 6–12 years with ADHD, ASD, or typical development (TD) using parental questionnaires. ANOVA and linear regression analyses were adjusted for age and sex. Children with ADHD and ASD showed more sleep problems (63.6 and 64.7%, vs 25.1% in TD) and shorter sleep duration than controls, while differences between ADHD and ASD were not significant. Sleep hygiene was worse in ADHD and ASD compared to TD, however, the association of worse sleep hygiene with more sleep problems was only significant in ASD and TD. There was a significant association of access to electronic media with sleep problems only in typically developing controls. Chronotype did not differ significantly between groups, but evening types were associated with sleep problems in ADHD and TD. Associations of greater anxiety/depression with more sleep problems were shown in ADHD and TD; however, anxiety/depression did not moderate the effects of chronotype and sleep hygiene. We conclude that sleep problems are highly prevalent in ADHD and ASD, but are differentially related to chronotype and sleep hygiene. In ASD, sleep problems are related to inadequate sleep hygiene and in ADHD to evening chronotype, while in TD both factors are important. Clinical implications are discussed.

**Electronic supplementary material:**

The online version of this article (doi:10.1007/s00787-017-1025-8) contains supplementary material, which is available to authorized users.

## Introduction

Sufficient and healthy sleep has proven to be important for developing children and adolescents [1]. Sleep disturbances in children can lead to emotional and behavioral problems, as well as to cognitive deterioration within the executive function domain, affecting functions such as mental flexibility, cognitive inhibition, and working memory [2]. The negative effects of impaired sleep are not confined to the period during which the individual child suffers from the disordered sleep, but seem to have a structural impact on development with residual negative effects on cognition and behavior existing even years after the sleep problems disappeared [3, 4]. Since sleep disorders occur very frequently in attention-deficit/hyperactivity disorder (ADHD) and autism spectrum disorders (ASD), and may worsen or mimic problem behavior related to ADHD and ASD [5, 6], it is important to gain better insight into etiology, prevention and intervention of sleep problems in those populations.

### Sleep problems, chronotype and circadian rhythm in ADHD and ASD

Many body functions, such as the sleep/wake cycle, variations in body temperature and release of hormones such as cortisol and melatonin show a 24-h cycle known as circadian rhythm. The suprachiasmatic nuclei in the brain, also called the ‘biological clock’, regulate these circadian processes. In a significant part of the population (~30%) those internal circadian rhythms are relatively advanced or delayed with respect to the clock time [7]. Those individuals are commonly referred to by ‘morning types’ or ‘evening types’, and the term used to denote this fairly stable trait is ‘chronotype’. Both genetic variations in biological clock genes and environmental influences, such as the light/dark rhythm, contribute to the distribution of chronotypes in a given population. Particularly children with a high preference for eveningness (evening chronotypes) experience a discrepancy between social time and internal biological time, which is called ‘social jetlag’. During schooldays they show a significantly reduced sleep length due to their inability to fall asleep at night, and they struggle to wake up in the morning before the circadian pacemaker exerts its activating action on the psychophysiological arousal system [8]. Recent research has shown that children and adolescents with evening preference demonstrate increased rates of behavioral and emotional problems, even suicidality, and substance use compared with intermediate and morning groups [8–11]. Given the strong association of chronotype with behavioral and emotional problems the issue is particularly important in child psychiatry.

In recent years, evidence has accumulated that ADHD is linked with delayed sleep-wake rhythms [12]. Sleep problems are reported in 50–80% of children with ADHD, which refers primarily to sleep onset insomnia [5], and daytime sleepiness [13]. To compare, in the general child population the prevalence of sleep problems is around 25% [14]. Adults with ADHD more often show an extreme evening chronotype [15–17], and 70% of adults with ADHD report difficulties with waking up in the morning and getting out of bed [18]. Studies on chronotype in children with ADHD are scarce [19]. Gruber and colleagues [20] assessed chronotype in 26 children with ADHD and 49 typically developing controls aged 7–11 years by means of the Child morning–evening preference scale. The results showed that children with ADHD showed a stronger evening tendency compared to children in the control group. Further, evening orientation was associated with subjectively and objectively assessed delay in sleep onset.

ASD is also frequently accompanied by co-morbid sleep disruption. Research suggests that about 40–80% of individuals with an ASD experience sleep problems [21]. The most prevalent sleep problems are sleep onset difficulties, longer sleep latency, earlier wake-up time and more night awakenings [22, 23]. Studies have shown abnormalities in melatonin production and rhythm [24, 25], which is indicative of dysregulated biological clock rhythms, although a recent study did not replicate those findings [26]. It remains unknown whether sleep problems in ASD are related to an evening chronotype.

### Sleep hygiene in ADHD and ASD

Parenting related factors associated with a child’s sleep are called ‘sleep hygiene’ and play an important role in sleep development and sleep problems, particularly in early childhood [27]. Sleep hygiene facilitates good sleep quality, longer sleep duration and reduces sleepiness during the day. An inconsistent bedtime routine, delayed or irregular bedtimes, television in the bedroom and caffeinated drinks just before bedtime have a negative influence on sleep and can precipitate or perpetuate sleep disturbances [28]. One currently important issue related to sleep hygiene is the access to electronic media devices, such as computers, tablets, smartphones and mobile phones by children in the evening. Adolescents with more media devices in their bedroom showed a shorter sleep duration and experienced more sleepiness during the day than adolescents with less media devices in their bedroom [29]. A study in The Netherlands in children aged 4–13 years showed that TV viewing and computer use (in general, i.e. not specifically in the evening) were associated with shorter sleep duration [30]. Another study in 6–16-year-old children showed that short sleep was associated with having a bedroom TV, spending more than 2 h a day at the TV or the computer, being tired in school, and having difficulties both in waking up and in sleeping [31].

Recent studies showed that children 8–18 years with ASD spent more hours per day playing video games and had higher levels of problematic video game use compared with typically developing siblings. In contrast, children with ASD spent less time using social media or socially interactive video games [32]. Furthermore, although bedroom media access was associated with less time spent sleeping per night, in ADHD, ASD, and typically developing children aged 8–17 years, the association was strongest for the ASD group [33].

Studies of sleep hygiene in children with developmental psychopathology are scarce, despite the evidence for its etiological role in sleep problems in the general child population. A study in children aged 6–12 years with ADHD revealed no differences in sleep hygiene between children with ADHD with and without sleep disorders [34], however, assessments of media use were not included. On the contrary, a recent study showed that improvement of sleep of children with ADHD via a sleep hygiene intervention resulted in positive changes of sleep and behavior [35]. Furthermore, a recent study in children with ASD aged 2–10 years showed that parent-based sleep education led to improvement of sleep onset [36]. These results indicate that sleep hygiene needs to be further explored in children with developmental disorders.

Previous studies have shown that ADHD and ASD are often accompanied with anxiety and depression problems [37]. Both anxiety and depression are associated with sleep disorders in children [38], and studies have demonstrated that anxiety and depression comorbidity affect sleep disturbances both in ADHD [39] and ASD [40]. It remains unknown whether in ADHD and ASD, associations of sleep disturbances with chronotype and sleep hygiene might be different for children with comorbid anxiety and depression than for children without those internalizing behavioral problems.

### Current study

The present study aimed to compare sleep patterns of children with ADHD, children with ASD and typically developing controls. Moreover, the association of sleep problems with sleep hygiene and chronotype was investigated in these different groups. Furthermore, it was investigated whether sleep problems in ADHD and ASD were related to comorbid mood and anxiety problems and to what extent associations of sleep disturbances in ADHD and ASD with chronotype and sleep hygiene were moderated by comorbid anxiety and depression. As described above, the ADHD and ASD populations are both characterized by increased prevalence rates of sleep disturbances; particularly sleep onset problems are common. Therefore, comparison of these two clinical samples and a typically developing control group might provide better insight into whether particular sleep patterns are disorder-specific. Based on the literature the following a priori hypotheses were formulated: (1) parents of children with ADHD and ASD will report a higher prevalence of sleep problems compared to typically developing controls, particularly sleep onset problems, a delayed sleep onset and awake time. There were no specific hypotheses regarding differences in prevalence rates between ADHD and ASD. (2) Children diagnosed with ADHD and ASD will show increased eveningness (i.e. more evening chronotypes) compared to normal controls [20]. There was no hypothesis with respect to the differences between clinical groups. (3) With regards to sleep hygiene, there were no hypotheses for ADHD and ASD due to a paucity of extant data from previous studies. Although some previous studies showed relationships of media use and sleep in ASD, the present study includes elementary school aged children instead of adolescents, which might result in different findings. (4) Sleep problems in typically developing controls will be predicted by both chronotype and sleep hygiene, whereas in ADHD a predictive value of chronotype is expected, while the role of sleep hygiene is unclear. For ASD, we had no specific hypotheses concerning the predictive value of chronotype and sleep hygiene on sleep problems. (5) Sleep problems in ADHD and ASD are related to comorbid mood and anxiety problems. There were no a priori expectations regarding moderation effects of anxiety and depression on the association of sleep disturbances with chronotype and sleep hygiene.

## Methods

### Participants and procedures

There were two methods of recruitment. First, parents were approached of children aged 6–12 years attending the department for behavior problems or the department for autism spectrum disorders at an academic child psychiatry outpatient clinic in The Netherlands (De Bascule). Children were diagnosed by experienced psychologists or psychiatrists according to DSM-IV, based on semi-standardized procedures and after multidisciplinary consultation. At the time of recruitment, assessment and intervention protocols for sleep disturbances were lacking at the outpatient clinics. All children received regular health care at the institution at the time of recruitment. Second, parents of typically developing children were recruited from 12 regular primary schools in The Netherlands. All parents received an information letter including an informed consent form and a stamped return envelope. In case of no response after 3 weeks, parents received a reminder by telephone. After having provided written informed consent, parents received a link to a digital questionnaire via email. Alternatively, they received the questionnaire on paper upon request.

A total of 383 parents gave informed consent, 116 from the outpatient clinic (response rate 16.6%) and 267 via primary schools (10.0%). Parents of 24 children recruited via primary schools reported a childhood psychiatric diagnosis (ADHD *n* = 12; ASD *n* = 8; other *n* = 4). The information was obtained with a single written question to parents whether the child had previously been diagnosed with a psychiatric disorder (e.g. ADHD, autism or anxiety disorder) by a psychologist, psychiatrist, physician or pediatrician after diagnostic assessment. Those children were excluded from participation. One child was excluded from the outpatient group due to missing data. The subsequent sample consisted of a total of 358 participants: 115 children with a diagnosed psychiatric disorder (81.7% boys; mean age 9.7 years, SD 1.8) and 243 typically developing controls (48.6% boys; mean age 8.7 years, SD 2.1). The outpatient group was split up based on diagnosis into a group with ADHD (*n* = 44), ASD (*n* = 68), and a group with other diagnoses (*n* = 3) which was excluded from this study, leaving a final number of 112 participants in the psychiatric group. 4 out of 44 children (9.1%) with a primary ADHD diagnosis were also diagnosed with ASD, and 22 (50.0%) with another psychiatric disorder. Of the 68 children with a primary ASD diagnosis, 16 (23.5%) had a co-morbid ADHD diagnosis and 7 (10.3%) another psychiatric diagnosis. Use of melatonin was 16% in children with a primary diagnosis of ADHD and 10% in ASD, while methylphenidate was used in 79% of children with a primary diagnosis of ADHD and in 32% with ASD. An average educational level score of the parents served as indicator of socioeconomic status. This score was based upon the scores of both the father and the mother on a 5-category response scale: (a) no education, (b) primary, (c) lower secondary, (d) higher secondary, and (e) tertiary. Seventy-four percent of the parents in the ADHD group had completed at least higher secondary school, whereas that was 84% in the ASD and typical control group (Table 1).

**Table 1:** Demographics in ADHD, ASD, typically developing controls

	ADHD (*N* = 44)	ASD (*N* = 68)	Control (*N* = 243)
Age (years)	9.8 (SD 1.6)	9.6 (SD 1.9)	8.7 (SD 2.1)
Sex (% boys)	70.5%	89.7%	51.9%
SES^a^			
No education	0%	0%	1%
Primary education	4%	0%	1%
Lower secondary education	21%	13%	13%
Higher secondary education	27%	26%	37%
Tertiary education	47%	59%	47%
Psychiatric co-morbidity	ASD = 4 (9.1%)	ADHD = 16 (23.5%)	None
	Other = 22 (50.0%)	Other = 7 (10.3%)	
	None = 18 (40.9%)	None = 42 (61.8%)	

^a^ Familial socioeconomic status, defined by parental educational level

### Instruments

#### Behavioral problems

The child behavior checklist (CBCL) is a parent-report questionnaire containing 113 items assessing a broad range of emotional and behavioral problems over the past 6 months in children aged 6–18 years [41, 42]. The CBCL consists of 113 items that are rated as not true; somewhat or sometimes true; very true or often true. Several scores can be derived from the questionnaire, such as internalizing and externalizing problems (broadband scales), total problems, and several subscales clustering specific problems (narrowband scales). In the present study, raw scores on the anxious/depressed scale and standardized T-scores (by age and sex) of the two broadband scales and the total problem scores were used (higher scores, more problems). The Dutch translation of the CBCL showed good reliability and validity in the Dutch population [42].

#### Sleep problems

Sleep was assessed with the Sleep Disturbance Scale for Children (SDSC), a well-validated and standardized parental report questionnaire [43]. The SDSC assesses sleep behavior of children and adolescents (ages 6–15 years). Parents were instructed to consider their child’s sleep during the past 6 months. The SDSC consists of 26 items with a 5-point Likert-type scale (never to always). Scores on six subscales can be derived: disorders of initiating and maintaining sleep (DIMS); sleep breathing disorders (SBD); disorders of arousal (sleepwalking, sleep terrors) (DA); sleep–wake transition disorders (SWTD); disorders of excessive somnolence (DES); and sleep hyperhydrosis (falling asleep sweating and night sweating) (SHY). A total sleep problem score (TSP) was derived by summing all subscale scores, yielding a continuous variable ranging from 26 to 130 with higher scores representing a higher frequency of sleep problem symptoms. To evaluate the prevalence of sleep problems, the total sleep problem score was also dichotomized at 39 points, with <39 points no sleep difficulties and >39 sleep difficulties as suggested by Bruni et al. [43]. The internal consistency was *α* = 0.79 and test–retest reliability *r* = 0.71 over a 6-month interval [43].

#### Sleep patterns

Sleep duration was assessed using questions where parents had to state the time when the child usually went to bed (bedtime), the time it took the child to fall asleep [sleep onset latency (SOL); in minutes], and the time the child usually woke up in the morning (awake time) in the last months, all for weekdays and weekends separately [8]. From these data, sleep onset time was calculated (sleep onset latency added to bedtime) and sleep duration per night (period from sleep onset until morning awake time) separately for weekdays and weekends [8]. Because of age differences in sleep duration, standardized *z*-scores per age group were calculated and used for the analyses.

#### Chronotype

Chronotype of the children was assessed with a Dutch translation of the Children’s Chronotype Questionnaire (CCTQ) [44] completed by parents. The morningness–eveningness subscale consists of 10 statements about the child’s time-of-day preference for certain activities and about waking in the morning, based on the preceding weeks. Scores are summed, yielding a continuous variable ranging from 10 to 48, with higher scores indicating increased eveningness. The continuous variable Total CCTQ score was used in the association analyses. Psychometric properties of the original CCTQ were shown to be adequate [44]. Parent ratings on the CCTQ were associated with melatonin onset in toddlers [45]. Cronbach’s alpha of the Dutch version was 0.78 [8].

#### Sleep hygiene

Sleep hygiene was assessed using the Children’s Sleep Hygiene Scale (CSHS) [46, 47]. The CSHS comprises 25 short questions about sleep hygiene with a 6-point response scale (1: never; 6: always) to be completed by the parents. A total sleep hygiene score was derived by summing all item scores (range 25–150, higher score indicating worse sleep hygiene). Cronbach’s alpha of the Dutch version was 0.76 [47].

#### Electronic media

Parents were asked to report whether their children used different types of media in the last hour before bedtime, and whether different types of media were active in the bedroom of their child, from the moment he/she lied in bed to sleep, similar to previous studies [32, 33]. There were six types of electronic media: internet, television, pc games, social media, texting, telephone. Answers had to be given on a 6-point scale: always (each day), often (3–5 times per week), sometimes (1–2 times per week), rarely (1–2 times per month), never, do not know. First all items where recoded to 5-point scale items, leaving the option ‘do not know’ out. Subsequently, all scores were reversed (higher score, more frequent use of media). Then two composite scores were derived, one for media use in the hour before going to bed (6 items) and one for media use while in bed (3 items), and a total score for media use (9 items).

### Statistical analysis

Analyses of variance were used to compare the ADHD, ASD and control group on several variables, such as sleep problems, sleep patterns, chronotype score, sleep hygiene, social media use, and behavior problems, controlled for age and sex. Hierarchical Linear Regression Analyses were used to investigate prediction of sleep problems by chronotype, sleep hygiene, electronic media use (model 2), over age and sex (model 1). All dependent variables were centered at their mean. When the effects of chronotype, sleep hygiene or electronic media use on sleep problems appeared significant in model 2, interaction effects were analyzed of significant predictor(s) with anxiety/depression (model 3). Statistical Package for Social Science version 21.0 was used to analyze the data. A *p* value of <0.05 was considered as statistically significant.

## Results

### Demographic and clinical characteristics

There was a significant difference between the three groups in age, *F*(3,354) = 6.62, *p* < 0.001. Both the ADHD group (*M* = 9.8, SD = 1.6 years) and the ASD group (*M* = 9.6, SD = 1.9 years) were significantly older than the control group (*M* = 8.7, SD = 2.1 years). A significant between-group difference was found in male:female ratio, with a nearly equal distribution within the control group (51.9% boys) and an overrepresentation of boys in the clinical groups (ADHD, 70.5% boys; ASD, 89.7% boys) (*p* < 0.001).

Results from the CBCL showed that the three groups differed significantly on the anxious/depressed scale, *F*(3,344) = 38.15, *p* < 0.001, internalizing problems, *F*(3,344) = 40.72, *p* < 0.001, externalizing problems, *F*(3,344) = 54.54, *p* < 0.001 and the Total Behavioral Problems score, *F*(3,344) = 87.69, *p* < 0.001. The Anxious/Depressed score was higher in ASD (7.69, SD = 5.04) than in ADHD (5.04, SD = 4.37) (*p* < 0.001), and both scores were higher than for controls (2.92, SD = 3.13), (both *p* < 0.001). The ASD group (*M* = 64.71, SD = 8.63) scored higher on internalizing problems than the ADHD group (*M* = 57.33, SD = 10.67) (*p* < 0.001), which scored higher than the control group (*M* = 50.19, SD = 10.48) (*p* < 0.001). The ADHD and ASD groups both showed higher scores for externalizing problems (ADHD, *M* = 62.91, SD = 11.15; ASD, *M* = 62.47, ASD = 10.24) and the total score of behavioral problems (ADHD, *M* = 64.30, SD = 8.59; ASD, *M* = 67.51, SD = 6.82), compared to the control group (externalizing, *M* = 48.76, SD = 9.47; Total, *M* = 50.10, SD = 9.63).

### Group comparisons

#### Sleep problems

Significant between group differences were found for DIMS, *F*(3,352) = 19.62, *p* < 0.001, as well as for DA, *F*(3,351) = 3.85, *p* = 0.01, SWTD, *F*(3,352) = 7.94, *p* < 0.001, DES, *F*(3,351) = 9.58, *p* < 0.001, SHY, *F*(3,352) = 4.18, *p* < 0.01, and TSP, *F*(3,352) = 19.15, *p* < 0.001 (Table 2) (all analyses controlled for age and sex). For DA, the ASD group had a significant higher score than the control group. For DIMS, SWTD, DES, SHY, and TSP the ADHD group and the ASD group both had significantly higher scores than the control group. The ADHD and the ASD group did not differ significantly from each other.

**Table 2:** Differences in sleep problems between ADHD, ASD, and controls

	ADHD (*N* = 44)	ASD (*N* = 67)	Control (*N* = 243)	*p*
DIMS	11.05 (4.13)	12.06 (3.69)	8.74 (2.78)	ADHD and ASD **>** Control***
SBD	4.20 (1.46)	4.25 (1.69)	3.98 (1.17)	NS
DA	4.02 (1.49)	4.25 (1.52)	3.70 (1.12)	ASD **>** Control**
SWTD	10.91 (4.01)	10.62 (3.79)	9.00 (2.59)	ADHD and ASD **>** Control***
DES	9.00 (3.20)	8.91 (2.73)	7.44 (2.06)	ADHD and ASD **>** Control***
SHY	3.75 (2.16)	3.71 (1.94)	2.97 (1.43)	ADHD > Control* and ASS > Control**
TSP	42.93 (10.99)	43.74 (9.79)	35.83 (7.22)	ADHD and ASD **>** Control***
Prevalence^a^	28 (63.6%)	44 (64.7%)	61 (25.1%)	ADHD and ASD > Control***

Values are means and standard deviations

*DIMS* Disorders of initiating and maintaining sleep, *SBD* sleep breathing disorders, *DA* disorders of arousal, *SWTD* sleep-wake transition disorders, *DES* disorders of excessive somnolence, *SHY* sleep hyperhidrosis, *TSP* total sleep problem score

*NS* not significant (*p* > 0.05)

* Significant at the 0.05 level

** Significant at the 0.01 level

*** Significant at the 0.001 level

^a^ Prevalence: total score >39

As for the total SDSC score (TSP), results showed that the ADHD group and the ASD group both had higher scores than the control group, *F*(3,352) = 15.84, *p* < 0.001, while the ADHD and ASD group did not differ significantly from each other. Further analyses of sleep problem prevalence rate (score >−39) showed that both the ADHD group (63.6%) and the ASD group (64.7%) had a higher prevalence of children with high sleep disturbance scores compared to controls (25.1%) (*p* < 0.001).

#### Sleep patterns

Group comparisons of bedtime, sleep onset latency, sleep onset time, awake time, and sleep duration during weekdays and weekends can be found in Table 3. On weekdays, sleep onset latency differed significantly between the three groups, *F*(3,348) = 11.63, *p* < 0.001, as well as sleep onset time, *F*(3,348) = 5.00, *p* < 0.001, awake time, *F*(3,348) = 3.44, *p* < 0.05, and sleep duration, *F*(3,346) = 8.33, *p* < 0.001 (all analyses controlled for age and sex). The ADHD group went to bed (20:36, SD = 0:44) significantly later than the control group (20:15, SD = 0:44) (*p* < 0.05). The ASD group woke up (06:52, SD = 0:33) significantly earlier than the control group (07:03, SD = 0:24) (*p* < 0.05). Sleep onset time was significantly later in ADHD (21:16, SD = 1:07) and ASD (21:04, SD = 0:55) compared to controls (20:35, SD = 0:48) (*p* < 0.001). The ADHD group (584 min, SD = 65) and ASD group (588 min, SD = 54) both showed a significantly shorter sleep duration than the control group (628 min, SD = 48) (*p* < 0.001). The ADHD group and the ASD group did not differ from each other on any of the sleep patterns (corrected for differences in age and sex).

**Table 3:** Differences in sleep patterns between ADHD, ASD, and controls

	ADHD (*N* = 44)	ASD (*N* = 67)	Control (*N* = 240)	*p*
Week days				
Bedtime	20:36 (0:44)	20:30 (0:46)	20:15 (0:44)	ADHD **>** Control*
Sleep onset latency (min)	40 (42)	34 (26)	20 (18)	ADHD and ASD **>** Control**
Sleep onset time	21:16 (1:07)	21:04 (0:55)	20:35 (0:48)	ADHD and ASD **>** Control**
Awake time	07:00 (0:31)	06:52 (0:33)	07:03 (0:24)	ASD **<** Control*
Sleep duration (min)	584 (65)	588 (54)	628 (48)	ADHD and ASD **<** Control**
Weekends				
Bedtime	21:17 (1:08)	21:04 (1:03)	21:03 (0:58)	NS
Sleep onset latency (min)	32 (20)	28 (25)	15 (14)	ADHD and ASD **>** Control**
Sleep onset time	21:49 (1:21)	21:32 (1:05)	21:18 (0:58)	ADHD **>** Control*
Awake time	07:38 (1:08)	07:36 (1:11)	07:54 (0:57)	NS
Sleep duration (min)	589 (58)	604 (58)	636 (51)	ADHD and ASD **<** Control**

Values are means and standard deviations

Analyses corrected for differences in age and sex

*NS* not significant (*p* > 0.05)

* Significant at the 0.05 level

** Significant at the 0.001 level

On weekends, sleep onset latency differed significantly for the three groups, *F*(3,348) = 5.50, *p* = 0.001, as well as sleep onset time, *F*(3,348) = 2.97, *p* < 0.05, and sleep duration, *F*(3,346) = 6.18, *p* < 0.001. SOL was significantly longer in the ADHD (32 min, SD = 20) and ASD group (28 min, SD = 25) than in the control group (15 min, SD = 14) (*p* < 0.001), while sleep duration was shorter in ADHD (589 min, SD = 58) and ASD (604 min, SD = 58) compared to controls (636 min, SD = 51) (*p* < 0.001). The ADHD group slept in significantly later (21:50, SD = 1:21) than the control group (21:18, SD = 0:58) (*p* < 0.05). There were no significant differences between the ADHD group and the ASD group.

#### Chronotype

Total CCTQ score did not differ significantly between the three groups, *F*(3,347) = 2.04, *p* = 0.11, after controlling for differences in age and sex: ADHD *M* = 27.85, SD = 7.37; ASD *M* = 27.99, SD = 6.79; controls *M* = 25.90, SD = 5.89.

#### Sleep hygiene and electronic media use

Sleep hygiene differed significantly between the three groups, *F*(3,347) = 7.21, *p* < 0.001, after adjustment for age and sex. The ADHD group (*M* = 52.53, SD = 10.19) and the ASD group (*M* = 50.33, SD = 9.46) both showed a significantly higher sleep hygiene score (i.e. worse sleep hygiene) than controls (*M* = 46.00, SD = 7.48). Group effects at item level are presented as Electronic Supplementary Material.

Group differences in total electronic media were borderline significant, *F*(2,351) = 2.58, *p* = 0.077. The control group showed a significantly more frequent use of electronic media before bedtime (*M* = 12.21, SD = 3.45), than the ASD group (*M* = 11.99, SD = 3.73) (*p* = 0.024) (Table 4).

**Table 4:** Differences in social media use between ADHD, ASD, and controls

	ADHD (*N* = 44)	ASD (*N* = 67)	Control (*N* = 243)	*p*
Before bedtime	12.92 (3.97)	11.99 (3.73)	12.21 (3.45)	ASD **<** Control*
In bed	3.48 (1.83)	3.13 (0.62)	3.36 (1.45)	NS
Total	16.40 (5.18)	15.12 (3.92)	15.57 (4.26)	ASD **<** Control*

Values are means and standard deviations

Analyses corrected for differences in age and sex

*NS* not significant (*p* > 0.05)

* Significant at the 0.05 level

### Predicting sleep problems in ADHD, ASD and controls

Linear regression analyses were used to investigate the predictive value of chronotype, sleep hygiene, and electronic media use on sleep problems, over age and sex. As can be seen in Table 5, the second model in ADHD explained 28.6% of the variance [*R* ^2^ = 0.286, *F*(5,48) = 3.84, *p* = 0.005]. In ADHD, evening chronotype of the child significantly predicted more sleep problems, Beta = 0.471, *p* = 0.002. For ASD, the second model explained 15.6% of the variance [*R* ^*2*^ = 0.156, *F*(5,64) = 2.36, *p* = 0.05]. Worse sleep hygiene predicted more sleep problems in ASD, Beta = 0.362, *p* = 0.01. The second model for the typically developing controls explained 18.3% of the variance [*R* ^2^ = 0.183, *F*(5,232) = 10.4, *p* < 0.001]. Results showed that evening chronotype, Beta = 0.317, *p* < 0.001, inadequate sleep hygiene, Beta = 0.312, *p* < 0.001, and electronic media use, Beta = 0.151, *p* < 0.05, predicted more sleep problems in the controls.

**Table 5:** Hierarchical regression analysis for variables predicting sleep problems in ADHD, ASD, and controls

ADHD	Model 1			Model 2			Model 3		
Variable	B	SE B	Beta	B	SE B	Beta	B	SE B	Beta
Age	−0.138	0.823	−0.023	−1.37	0.871	−0.226	−1.32	0.848	−0.218
Sex	4.96	2.87	0.235	0.144	2.85	0.007	0.136	2.74	0.006
Chronotype				0.615	0.185	0.471**	0.638	0.188	0.488**
Sleep hygiene				0.187	0.128	0.190	0.161	0.124	0.163
Electronic media				−0.135	0.260	−0.072	−0.156	0.250	−0.083
Anx/depr							0.637	0.259	0.292*
Chronotype × anx/depr							−0.006	0.036	−0.023
*R* ^2^		0.056			0.286			0.369	
*F* for change in *R* ^2^		1.51			5.14**			3.03	

ASD	Model 1			Model 2			Model 3		
Variable	B	SE B	Beta	B	SE B	Beta	B	SE B	Beta
Age	−0.612	0.647	−0.117	−0.646	0.699	−0.123	−0.574	0.686	−0.110
Sex	0.101	4.06	0.003	0.755	3.85	0.023	0.527	3.78	0.016
Chronotype				0.150	0.189	0.101	0.181	0.185	0.121
Sleep hygiene				0.379	0.142	0.362**	0.174	0.174	0.166
Electronic media				0.278	0.355	0.111	0.293	0.354	0.117
Anx/depr							0.416	0.262	0.208
Sleep hygiene × anx/depr							0.022	0.024	0.154
*R* ^2^		0.014			0.156			0.218	
*F* for change in *R* ^2^		0.468			3.59*			2.46	

Controls	Model 1			Model 2			Model 3		
Variable	B	SE B	Beta	B	SE B	Beta	B	SE B	Beta
Age	0.157	0.224	0.046	−0.434	0.273	−0.127	−0.599	0.256	−0.175*
Sex	−0.164	0.950	−0.011	−0.684	0.872	−0.047	−0.431	0.817	−0.030
Chronotype				0.389	0.090	0.317**	0.358	0.083	0.292**
Sleep hygiene				0.307	0.065	0.312**	0.201	0.070	0.204**
Electronic media				0.253	0.124	0.151*	0.129	0.129	0.077
Anx/depr							0.886	0.137	0.382**
Chronotype × anx/depr							−0.016	0.018	−0.057
Sleep hygiene × anx/depr							0.010	0.022	0.032
Electronic media × anx/depr							0.005	0.034	0.010
*R* ^2^		0.002			0.183			0.313	
*F* for change in *R* ^2^		0.276			17.05**			10.782**	

* *p* < 0.05, ** *p* < 0.01

Subsequent analyses in ADHD [model 3, *R* ^2^ = 0.369, *F*(7,46) = 3.84, *p* = 0.05] revealed a significant and positive main effect of chronotype, Beta = 0.488, *p* = 0.001, and anxiety/depression, Beta = 0.292, *p* = 0.018. There was no significant chronotype × anxiety/depression interaction in ADHD. Analyses for the ASD group showed neither a main effect of anxiety/depression, nor an interaction of sleep hygiene × anxiety/depression. In typically developing controls [model 3, *R* ^2^ = 0.313, *F*(9,228) = 11.52, *p* < 0.001], significant main effects were found for chronotype, Beta = 0.292, *p* < 0.001, sleep hygiene, Beta = 0.204, *p* = 0.004, and anxiety/depression, Beta = 0.382, *p* < 0.001, however, interaction effects of anxiety/depression with either chronotype, sleep hygiene, or electronic media use were not significant.

## Discussion

This study aimed to compare sleep problems and sleep patterns of children with ADHD, ASD, and typically developing controls, and to investigate within those groups the association of sleep problems with sleep hygiene and chronotype. As expected we found significant differences in sleep patterns between both clinical groups and the typically developing controls. Sleep onset during weekdays was around 30–45 min later in ADHD and ASD than in controls. ASD youth woke up earlier than controls despite a later sleep onset time, which is in line with previous reports of frequent early morning awakenings [48], but which contradicts findings in other studies [6]. Whether the early rise times are due to biological differences in sleep patterns and/or environmental constraints on sleep related to the diagnosis (e.g., awoken earlier to allow more time for morning routines) remains unknown. Sleep duration was significantly shorter—around 45 min—in ADHD and ASD compared to controls. Along this line, parents indicated that children with ADHD and ASD both suffered more frequently than controls from sleep problems. That was the case for all types of sleep problems in our study, except for sleep breathing disorders. Furthermore, disorders of arousal were more prevalent in ASD, but not in ADHD, compared to controls. Importantly, sleep problems were present in nearly two-third of the children with ADHD (64%) and ASD (65%), compared to 25% in controls. Those high prevalence rates correspond to previous findings [5, 6], and underline the importance of this issue in ADHD and ASD. Importantly, we found no differences in sleep patterns or sleep problems between the clinical groups. Thus, our results demonstrate that both psychiatric disorders are characterized by equally high prevalence rates and a broad spectrum of sleep problems.

As for the prediction of sleep problems, we found that sleep disturbances were differentially predicted by eveningness and sleep hygiene within the various groups. As expected, in normal controls both eveningness and sleep hygiene were associated with sleep disturbances. However, sleep disturbances in children with ADHD were only related to increased eveningness and not to sleep hygiene. Moreover, eveningness was of more predictive value for sleep problems in ADHD than in typically developing controls. That is in line with our hypotheses, since eveningness is associated with circadian rhythm markers [49, 50], and those markers have been found delayed in ADHD [15, 34, 51]. Furthermore, genetic studies showed Clock gene aberrances in ADHD [52, 53] and an absence of rhythmic expression in BMAL1 and PER2 clock gene products [15]. Higher prevalence rates of evening chronotype were also previously found in childhood ADHD [20] and adult ADHD [15–17]. Intriguingly, although we were able to replicate the association of sleep problems with increased eveningness in ADHD, and although sleep onset was delayed in ADHD, we did not find differences in chronotype scores between ADHD, ASD, and controls. It remains unknown how a delayed sleep onset in ADHD can be reconciled with the lack of chronotype findings. Possibly, delayed sleep onset was due to behavior differences in ADHD versus controls such as bedtime resistance [54], limit-setting problems or inadequate enforcement of bedtimes by caregivers [55], or staying up later due to finishing homework that took longer because of distractibility, rather than biological rhythm differences.

As for sleep hygiene, our results were in line with earlier findings in ADHD. Van der Heijden and colleagues [47] showed that children with ADHD and sleep onset insomnia did not differ in sleep hygiene from children with ADHD without sleep disturbances. Although the current data revealed no significant prediction of sleep problems by sleep hygiene in ADHD, we did find a reduced sleep hygiene level in ADHD compared to controls. This suggests that poorer levels of sleep hygiene in families with ADHD might be an epiphenomenon of a disorganized home environment, partly resulting from parental problems with inhibition of impulses, sustained attention, and consistency [56]. Another explanation is that the effects of sleep hygiene in ADHD are masked or suppressed by eveningness. In other words, because of the detrimental effects of chronotype on sleep problems in ADHD, the additional effects of inadequate sleep hygiene might appear insignificant.

As expected, and consistent with previous findings, higher levels of anxiety and depression problems predicted more sleep problems both in ADHD and in typically developing controls [38]. The negative effect of eveningness on sleep problems in ADHD was not moderated by anxiety and depression problems. In other words, the findings demonstrate that evening types among children with ADHD have a higher risk of sleep problems, irrespective of the presence of comorbid anxiety and depression problems. Similarly, the results showed that the negative effects of evening chronotype and inadequate sleep hygiene in typically developing controls were not moderated by anxiety and depression problems. Interestingly, sleep problems in ASD were not associated with anxiety and depression problems. A possible explanation is that a relatively strong positive association of sleep hygiene with anxiety/depression in ASD (*r* = 0.34, *p* = 0.002), led to a mutually diminishing effect in the final model. A recent study demonstrated a contradicting finding that children with ASD and comorbid anxiety were particularly predisposed to sleep problems [57]. More future research on the interplay of sleep hygiene, anxiety/depression, and sleep disturbances in ASD is needed.

As for ASD, the results revealed that sleep problems were not related to eveningness and that there were no differences in eveningness scores between ASD and normal controls. Those results indicate that although ASD might be associated with disturbances in melatonin production and rhythm [24], those melatonin disturbances may not affect sleep wake rhythm. That is in accordance with a recent study showing that children with ASD and insomnia who were responsive to a low dose of melatonin had relatively normal profiles of baseline endogenous melatonin and normal pharmacokinetic melatonin profiles after supplemental melatonin administration [26]. Thus, apparently, melatonin did not improve sleep problems in ASD through replacing endogenous melatonin or acting on the circadian system. It remains unknown what the behavioral correlates of the endogenous melatonin disturbances in ASD are, but evidence suggests that sleep problems in ASD are not associated with a delayed circadian system, but might be due to an inadequate sleep hygiene. Sleep hygiene intervention studies in the ASD population are scarce so far [36], however the current findings suggest that sleep hygiene improvement might be an effective intervention strategy in children with ASD. Nevertheless, the results showed that negative effects of inadequate sleep hygiene disappeared when the predictive model included anxiety and depressive problems. This contradicts the possible role of sleep hygiene, and emphasizes the importance for future studies to control for anxiety and depressive problems.

There was a significant association of access to electronic media with sleep problems in typically developing controls, but there were no significant differences in electronic media use between the psychiatric populations and the typically developing children, except for a more frequent use of electronic media before bedtime in the control group compared to the ASD group. Previous studies in children have found a frequent use of electronic media, particularly in ASD, and demonstrated associations of sleep and media use in ADHD, ASD and typically developing children [32, 33], however, those studies were conducted in adolescents. Of note, there are recent suggestions of a higher sensitivity for light in the ADHD population which might impact on the circadian system and therefore affect sleep wake rhythm [58]. We conclude that electronic media use might play a significant role in the etiology of sleep problems in elementary school aged children, however, more research is needed in children with ADHD and ASD.

There are several methodological strengths of this study. The inclusion of both an ADHD and ASD group enabled a comparison of clinical groups which provided the possibility to infer about the specificity of the various findings. As our data demonstrated, predictors of sleep problems varied for the different clinical and nonclinical groups. Other strengths are the diagnostic procedures of ADHD and ASD which were carried out by trained psychologists and psychiatrists according to DSM-IV and based on multidisciplinary consultation. Furthermore, well validated instruments were used to assess sleep, eveningness, and sleep hygiene.

However, our study also has several limitations. First, assessments were based on subjective parent reports. Although the instruments are well validated and commonly used in research, objective assessments of behavioral problems, sleep, and chronotype would have strengthened our findings. For instance, the assessment of electronic media use took place through parent report, however we cannot assure that parents were fully aware of the use of electronic media in bed by their children. Objective registration of electronic media use was not feasible in the current study, but is recommended for future studies. Furthermore, assessments of chronotype by means of objective measures such as actigraphy or dim light melatonin onset would have strengthened our study.

Second, this study includes only one single assessment. In order to infer conclusions about the etiological role of sleep hygiene and chronotype long term cohort studies are required. Third, we assessed chronotype through a questionnaire which usually shows high correspondence with objective circadian measures such as with endogenous melatonin, cortisol, or body temperature [59], however, the measures are not interchangeable. Where markers of circadian rhythm inform about the time of the circadian system, chronotype reflects the subjective individual experience of circadian preference and includes also the impact on daily life. The subjective nature of this measure therefore entails high ecological validity, but also requires caution as to conclusions about circadian disturbances. Fourth, we cannot exclude with certainty the presence of ADHD or ASD in the typically developing control group. Although 4.5% of the control group was excluded based on parental reports of a diagnosis of ADHD, and 3% of ASD, it is possible that there were children with ADHD or ASD in the control group who had not been formally assessed or diagnosed previously. A rigorous screening procedure with validated instruments would have provided more certainty that all children in the control group were indeed ‘typically developing’.

Finally, we cannot exclude the influence of medication use on the findings. Psychostimulants exert a negative effect on sleep and circadian rhythms [12], although this cannot explain the absence of differences in chronotype in the present study. Possibly, deteriorating effects on sleep and circadian rhythm taking place after commencement with stimulants were opposed by alleviating effects of melatonin treatment started because of the sleep disturbances.

To conclude, this study aimed to shed light on the potential role of chronotype and sleep hygiene in the etiology of sleep problems in ADHD, ASD and typical developing children. The study shows that both chronotype and sleep hygiene are associated with sleep problems in typically developing children, whereas in children with ADHD sleep problems are related to chronotype and in children with ASD only to inadequate sleep hygiene. Furthermore, the deteriorating role of anxiety and depressive problems in childhood sleep problems was corroborated in this study, however, negative effects of evening chronotype and inadequate sleep hygiene were not moderated by the presence of anxiety or depression.

The findings have implications for the clinical field since they corroborate that intervention and prevention programs of sleep disturbances should be tailored to different psychiatric diagnoses. As this study reveals, treatment strategies in ASD should contain a strong focus on sleep hygiene improvements, while interventions in ADHD should include methods to shift circadian preference, such as bedtime fading [60, 61], melatonin treatment [62], or early morning light therapy [63]. We do not suggest that sleep hygiene improvements are not relevant for children with ADHD. Individuals with ADHD who present with inadequate sleep hygiene might certainly profit from sleep hygiene improvements. This is particularly relevant since a great deal of children with ADHD have traits of ASD and vice versa. Sleep hygiene interventions should always be the first step in the management of sleep disturbances. Also, certain aspects of sleep hygiene interventions, such as a reduction of bright light in the late evening or avoidance of sleeping in over weekends, can have beneficial (phase-advancing) effects on evening chronotypes. Similarly, methods to shift circadian preference should be considered in children with ASD and traits of ADHD, particularly when sleep hygiene interventions do not seem to be sufficiently effective. Further replication studies are necessary to validate the findings of this association study.

## Electronic supplementary material

Below is the link to the electronic supplementary material.

Supplementary material 1 (DOCX 22 kb)
