# Supplementary material for: Sleep, chronotype, and sleep hygiene in children with attention-deficit/hyperactivity disorder, autism spectrum disorder, and controls
Source: Eur Child Adolesc Psychiatry. 2017 Jul 8;27(1):99–111. doi: 10.1007/s00787-017-1025-8 (PMC5799342; doi:10.1007/s00787-017-1025-8)
Supplement: Supplementary file 1 — Supplementary material 1 (DOCX 22 kb) [file 787_2017_1025_MOESM1_ESM.docx]

***Electronic Supplementary Material***

**Table 1. Differences in aspects of sleep hygiene between ADHD (0) and typically developing controls (1). Ordinal regression analyses, including sex and age.**

|  | Sleep hygiene* | Estimate | Std. Error | Wald | Sig. |
| --- | --- | --- | --- | --- | --- |
| 1 | Naps within 4 h before bedtime | -.776 | .639 | 1.477 | .224 |
| **2** | **Caffeine within 4 h before bedtime** | **.715** | **.316** | **5.120** | **.024** |
| 3 | Does things that are relaxing before bedtime | .220 | .275 | .638 | .424 |
| 4 | Drinks a lot of liquids before bedtime | -.148 | .289 | .263 | .608 |
| **5** | **Plays rough before bedtime** | **-.627** | **.286** | **4.792** | **.029** |
| 6 | Does things that are alerting before bedtime | .033 | .281 | .014 | .906 |
| 7 | Goes to bed at about the same time every day | -.467 | .302 | 2.388 | .122 |
| **8** | **Complains about being hungry at bedtime** | **-1.184** | **.289** | **16.779** | **.000** |
| **9** | **Does things in bed that keeps him/her awake** | **-1.096** | **.285** | **14.791** | **.000** |
| 10 | Goes to bed in the same place | .246 | .306 | .648 | .421 |
| 11 | Goes to bed feeling upset | -.378 | .359 | 1.112 | .292 |
| 12 | Goes to bed with worries | -.271 | .286 | .899 | .343 |
| 13 | Sleeps in a darkened room | .048 | .307 | .024 | .877 |
| 14 | Sleeps in a room that is too hot or too cold | -.021 | .311 | .004 | .947 |
| 15 | Sleeps in a room where there are loud noises | -.182 | .356 | .261 | .609 |
| **16** | **Sleeps in alone (in his/her own bed)** | **.915** | **.306** | **8.928** | **.003** |
| 17 | Sleeps in a room that is stuffy | -.241 | .311 | .602 | .438 |
| **18** | **Sleeps all or part of the night with someone else** | **-.633** | **.297** | **4.538** | **.033** |
| 19 | Sleeps in a bed that is comfortable | .600 | .465 | 1.664 | .197 |
| **20** | **Sleeps in a home where someone smokes** | **-1.106** | **.382** | **8.363** | **.004** |
| 21 | Has a calming bedtime routine | .166 | .274 | .366 | .545 |
| 22 | Uses bed for things other than sleep | -.472 | .280 | 2.844 | .092 |
| 23 | Put to bed after falling asleep | -.382 | .446 | .737 | .391 |
| 24 | Stays up past usual bedtime | -.371 | .290 | 1.637 | .201 |
| 25 | Gets out of bed about same time in morning | -.437 | .299 | 2.135 | .144 |

** Score 0: never - score 6: always*

**Table 2. Differences in aspects of sleep hygiene between ASD (0) and typically developing controls (1). Ordinal regression analyses, including sex and age.**

|  | Sleep hygiene | Estimate | Std. Error | Wald | Sig. |
| --- | --- | --- | --- | --- | --- |
| **1** | **Naps within 4 h before bedtime** | **-1.261** | **.591** | **4.557** | **.033** |
| **2** | **Caffeine within 4 h before bedtime** | **.906** | **.303** | **8.908** | **.003** |
| **3** | **Does things that are relaxing before bedtime** | **-.515** | **.255** | **4.072** | **.044** |
| 4 | Drinks a lot of liquids before bedtime | .269 | .267 | 1.012 | .314 |
| 5 | Plays rough before bedtime | -.511 | .263 | 3.771 | .052 |
| 6 | Does things that are alerting before bedtime | .359 | .258 | 1.935 | .164 |
| **7** | **Goes to bed at about the same time every day** | **-.728** | **.287** | **6.456** | **.011** |
| 8 | Complains about being hungry at bedtime | -.304 | .272 | 1.249 | .264 |
| **9** | **Does things in bed that keeps him/her awake** | **-.754** | **.261** | **8.385** | **.004** |
| 10 | Goes to bed in the same place | .217 | .279 | .605 | .437 |
| **11** | **Goes to bed feeling upset** | **-1.342** | **.303** | **19.624** | **.000** |
| **12** | **Goes to bed with worries** | **-1.239** | **.269** | **21.221** | **.000** |
| 13 | Sleeps in a darkened room | .439 | .270 | 2.647 | .104 |
| 14 | Sleeps in a room that is too hot or too cold | -.302 | .277 | 1.184 | .277 |
| 15 | Sleeps in a room where there are loud noises | -.228 | .322 | .503 | .478 |
| **16** | **Sleeps in alone (in his/her own bed)** | **.578** | **.288** | **4.032** | **.045** |
| 17 | Sleeps in a room that is stuffy | -.119 | .292 | .167 | .683 |
| 18 | Sleeps all or part of the night with someone else | -.319 | .281 | 1.295 | .255 |
| 19 | Sleeps in a bed that is comfortable | .676 | .428 | 2.501 | .114 |
| 20 | Sleeps in a home where someone smokes | .335 | .475 | .496 | .481 |
| 21 | Has a calming bedtime routine | -.400 | .255 | 2.470 | .116 |
| 22 | Uses bed for things other than sleep | -.209 | .256 | .666 | .415 |
| 23 | Put to bed after falling asleep | -.460 | .387 | 1.412 | .235 |
| 24 | Stays up past usual bedtime | .149 | .268 | .307 | .580 |
| 25 | Gets out of bed about same time in morning | -.239 | .274 | .763 | .382 |
